# Takayasu's arteritis is associated with HLA-B*52, but not with HLA-B*51, in Turkey

**DOI:** 10.1186/ar3730

**Published:** 2012-02-06

**Authors:** Ziver Sahin, Muge Bıcakcıgil, Kenan Aksu, Sevil Kamali, Servet Akar, Fatos Onen, Omer Karadag, Zeynep Ozbalkan, Askin Ates, Huseyin TE Ozer, Vuslat Yilmaz, Emire Seyahi, Mehmet A Ozturk, Ayse Cefle, Veli Cobankara, A Mesut Onat, Ercan Tunc, Nursen Düzgün, Sibel Z Aydin, Neslihan Yilmaz, İzzet Fresko, Yasar Karaaslan, Sedat Kiraz, Nurullah Akkoc, Murat Inanc, Gokhan Keser, F Aytul Uyar, Haner Direskeneli, Güher Saruhan-Direskeneli

**Affiliations:** 1 Department of Physiology, Istanbul University, Istanbul Faculty of Medicine, Capa 34093, Istanbul, Turkey; 2 Department of Rheumatology, Yeditepe University, Faculty of Medicine, Ankara Cad No:102/104, Kozyatagı 34752, Istanbul, Turkey; 3 Department of Rheumatology, Ege University, Faculty of Medicine, Ankara Asfalti Uzeri, Bornova 35100, Izmir, Turkey; 4 Department of Rheumatology, Istanbul University, Istanbul Faculty of Medicine, Capa 34093, Istanbul, Turkey; 5 Department of Rheumatology, Dokuz Eylül University, Faculty of Medicine, Dokuz Eylul Universitesi Saglık Yerleskesi, Inciraltı 35340, Izmir, Turkey; 6 Department of Rheumatology, Hacettepe University, Faculty of Medicine, Sihhiye 06100, Ankara, Turkey; 7 Department of Rheumatology, Ankara Numune Training and Research Hospital, Talatpasa Bulvarı, No:5, Altındag 06100, Ankara, Turkey; 8 Department of Rheumatology, Cukurova University, Faculty of Medicine, Balcalı, Sarıcam 01330, Adana, Turkey; 9 Department of Rheumatology, Istanbul University, Cerrahpaşa Faculty of Medicine, Kocamustafapaşa Cad. No: 124, Cerrahpaşa 34098, Istanbul, Turkey; 10 Department of Rheumatology, Gazi University, Faculty of Medicine, Besevler 06500, Ankara, Turkey; 11 Department of Rheumatology, Kocaeli University, Faculty of Medicine, Eski İstanbul Yolu, Umuttepe Yerleşkesi 41380, Kocaeli, Turkey; 12 Department of Rheumatology, Pamukkale University Faculty of Medicine, Üniversite Caddesi Kınıklı Kampüsü 20020, Denizli, Turkey; 13 Department of Rheumatology, Gaziantep University, Faculty of Medicine, Sahinbey Araştırma Hastanesi 27310, Gaziantep, Turkey; 14 Department of Rheumatology, Suleyman Demirel University, Faculty of Medicine, Doğu Kampüsü Morfoloji Binası Kat:2 32260, Isparta, Turkey; 15 Department of Rheumatology, Ankara University, Faculty of Medicine, Morfoloji Yerleşkesi, Sıhhıye 06100, Ankara, Turkey; 16 Department of Rheumatology, Marmara University, Faculty of Medicine, Mimar Sinan Caddesi, No: 41, Pendik 34890, Istanbul, Turkey

## Abstract

**Introduction:**

HLA-B*51 and HLA-B*52 are two close human leukocyte antigen (HLA) allele groups with minor amino acid differences. However, they are associated with two different vasculitides (HLA-B*51 in Behçet's disease and HLA-B*52 in Takayasu's arteritis (TAK)) and with major clinical and immunological differences. In this study, we aimed to screen a large cohort of TAK patients from Turkey for the presence of HLA-B*51 and HLA-B*52 as susceptibility and severity factors.

**Methods:**

TAK patients (*n *= 330) followed at a total of 15 centers were included in the study. The mean age of the patients was 37.8 years, and 86% were women. DNA samples from the patients and healthy controls (HC; *n *= 210) were isolated, and the presence of HLA-B*51 or HLA-B*52 was screened for by using PCR with sequence-specific primers.

**Results:**

We found a significant association of HLA-B*52 with TAK (20.9% vs HC = 6.7%, *P *= 0.000, OR = 3.7, 95% CI = 2.02 to 6.77). The distribution of HLA-B*51 did not differ between TAK patients and HCs (22.7% vs 24.8%, OR = 0.9, 95% CI = 0.60 to 1.34). The presence of HLA-B*52 decreased in late-onset patients (> 40 years of age; 12.0%, *P *= 0.024, OR = 0.43, 95% CI = 0.20 to 0.91). Patients with angiographic type I disease with limited aortic involvement also had a lower presence of HLA-B*52 compared to those with all other disease subtypes (13.1% vs 26%, *P *= 0.005, OR = 0.43, 95% CI = 0.23 to 0.78).

**Conclusions:**

In this study, the previously reported association of TAK with HLA-B*52 in other populations was confirmed in patients from Turkey. The functional relevance of HLA-B*52 in TAK pathogenesis needs to be explored further.

## Introduction

Takayasu's arteritis (TAK), also known as "pulseless disease," is a chronic granulomatous panarteritis characterized by the involvement of large vessels, especially the aorta and its major branches [1,2]. Although the etiology of TAK is still unknown, infectious agents, genetic factors and autoimmunity are thought to play a major role in its pathogenesis [3]. Cell-mediated autoimmunity has been implicated in the physiopathology of vascular cell injury in TAK. In addition to γδ T cells, natural killer (NK) cells and macrophages, tissue specimens from the aortas of TAK patients are infiltrated with T cells that have a restricted T-cell repertoire, which is typical of antigen-induced proliferation [4-6].

Evidences of genetic susceptibility to TAK have previously been demonstrated for SNPs of cytokine genes such as IL-2, IL-6 and IL-12 and the *NFKBIL1 *promoter region [7,8], but not with autoimmunity associated genes such as *PTPN22 *[9] and *PDCD1 *[10]. Genes encoding human leukocyte antigen (HLA) are highly polymorphic, show remarkable ethnic and geographic differences in allele and haplotype frequencies and are natural candidates for genetic susceptibility to immune and inflammatory diseases. Although some associations with class II alleles such as HLA-DRB*1301 have been reported, studies from mainly East Asian countries have demonstrated HLA-B*52 as the main risk factor in the HLA region [11,12]. However, this association has not been confirmed in North American and Arab populations [13,14].

HLA-B*51 and HLA-B*52 are two *HLA-B *allele groups with only two amino acid differences [15]. However, HLA-B*51 is associated with a phenotypically separate vasculitis, namely, Behçet's disease [16]. Previously, associations of both major HLA-B subtypes HLA-B*51 and HLA-B*52 with TAK have been shown only in an Indian population [17]. On the basis of this background, we screened patients with TAK in Turkey for the presence of HLA-B*51 and HLA-B*52.

## Materials and methods

The study was designed as a case-control study. We enrolled 330 patients with TAK (42 men and 288 women, mean age 38.9 ± 12.2 years) of mixed ethnic origin from Turkey. The patients were referred from 15 tertiary university and state hospital rheumatology centers in Turkey. Patients were classified according to the 1990 American College of Rheumatology criteria for TAK [18]. According to the angiographic classification scheme defined at the International Conference on Takayasu's Arteritis in Tokyo, 39.4% (*n *= 130) of the patients had type I vessel involvement, 6.4% had type IIa (*n *= 21), 2.7% had type IIb (*n *= 9), 3.9% had type III (*n *= 13), 4.5% had type IV (*n *= 15) and 43% had type V (*n *= 142) [19]. A surgical procedure was performed in 21.1% of the patients, with 11.6% done after immunosuppressive (IS) therapy. Patients with late-onset (> 40 years of age) disease was present in 23.8% of the patients. Corticosteroid treatment was given to 95% of the patients, standard first-line ISs (methotrexate, azatioprine, leflunomide or cyclophosphamide) were administered to 94.5% and second-line ISs (TNF antagonists) were given to 3.5%.

To evaluate HLA-disease phenotype associations, our study group proposed a consensus definition of "refractory disease" for TAK. Patients with "angiographic or clinical progression despite treatment" or any of the following characteristics were accepted to have "refractory" disease: corticosteroid dose > 7.5 mg/day after 6 months of treatment, despite the administration of conventional ISs (methotrexate, azatioprine, leflunomide or cyclophosphamide); new surgery due to persistent disease activity; frequent attacks (more than three yearly); or death associated with disease activity. On the basis of this definition, 27.4% (*n *= 114) of the patients with sufficient follow-up for analysis had refractory disease. Eighteen patients (5.9%) died during follow-up.

A total of 210 healthy blood donors (97 men and 113 women, mean age 32.2 ± 10.9 years) with the same mixed ethnic origin from Turkey were recruited for participation as healthy controls (HCs). All patients and controls were enrolled with the approval of the Marmara University Medical School Local Ethics Committee and provided their informed consent.

### Genotyping

For genotyping, cellular DNA was isolated from peripheral blood using standard procedures. For the determination of HLA-B*52 and HLA-B*51 alleles, DNA was amplified using the forward primers HLA 192 and HLA 193, respectively, and reverse primer HLA 216 (for both) for the specific product with another pair of primers was used as a control product [20]. PCR amplification was carried out in NH_4 _buffer with 1.3 mM MgCl_2_, 0.2 μM deoxyribonucleotide triphosphate, 0.5 μM of each primer, 60 ng of genomic DNA and 1 IU of *Taq *polymerase. The cycling parameters were as follows: an initial denaturation step of 2 minutes at 95°C; 5 cycles of 20 seconds at 95°C, 60 seconds at 64°C and 20 seconds at 72°C followed by 25 cycles of 20 seconds at 95°C, 60 seconds at 63°C and 20 seconds at 72°C; and a final extension step of 2 minutes at 72°C. Products were run on 1.5% agarose gel stained with ethidium bromide.

### Statistical analysis

HLA alleles in TAK patients were compared with those of the HCs and within the patient group. The strength of the association was expressed by the OR, and the statistical significance was examined by χ^2 ^test.

## Results

We found a significant association of HLA-B*52 with TAK (20.9% of patients (69 of 330) vs 6.7% of HCs (14 of 210), *P *= 0.000, OR = 3.7, 95% CI = 2.02 to 6.77). The association of HLA-B*52 with TAK was noted in women (21.5% vs 7.1%, *P *= 0.000, OR = 3.6, 95% CI = 1.66 to 7.79), but did not reach significance in men (16.7% vs 6.2%, *P *= 0.063). The distribution of HLA-B*51 did not differ between TAK patients and controls (22.7% of patients (75 of 330) vs 24.8% of HCs (52 of 210), *P *= 0.3, OR = 0.9, 95% CI = 0.60 to 1.34).

When we investigated HLA-B*52 presence according to the onset of disease, a decreased presence of HLA-B*52 was observed in late-onset patients (> 40 years of age) (early onset 24.2% vs late onset 12.0%, *P *= 0.024, OR = 0.43, 95% CI = 0.20 to 0.91) (Table 1). We then investigated whether the presence of HLA-B*52 affects the disease phenotype. Although we observed that the frequency of HLA-B*52 was significantly higher in all disease subtypes than in HCs, a lower presence of HLA-B*52 was present in patients with type I disease (13.1%) (*P *= 0.005, OR = 0.43, 95% CI = 0.24 to 0.78) compared to all other types of vessel involvement (types IIa through V, 26%). Although the finding did not reach statistical significance, patients defined as having refractory disease also seemed to have a stronger association with HLA-B*52 (refractory 27.4% vs nonrefractory 18.1%, *P *= 0.84, OR = 1.68, 95% CI = 0.97 to 2.93). No association of surgery and IS drug requirement after surgery with HLA-B*52 presence was present (surgery HLA-B*52 13.6% vs 21.6%, *P *= 0.092, OR = 0.51, 95% CI = 0.24 to 1.09; IS after surgery HLA-B*52 19.4% vs 21.6, *P *= 1.0). No association of HLA-B*51 presence was observed with gender, age, disease phenotype, treatment or any other clinical feature of TAK (Table 1).

**Table 1:** Distribution of HLA-B*51 and HLA-B*52 allele groups in healthy controls and Takayasu's arteritis patients^a^

Characteristics	*n*	HLA-B*52+ (%)	*P*	OR (95% CI)	HLA-B*51+ (%)	*P*
HC	210	14 (6.7)			52 (24.8)	
Men	97	6 (6.2)			23 (23.7)	
Women	113	8 (7.1)			29 (25.7)	
TAK	330	69 (20.9)	0.000	3.7 (2.02 to 6.77)	75 (22.7)	0.64
Men	42	7 (16.7)	0.063		12 (28.6)	0.53
Women	288	62 (21.5)	0.000	3.6 (1.66 to 7.79)	63 (21.9)	0.43
Age at onset						
< 40 years	240	58 (24.2)	0.024	0.43 (0.20 to 0.91)	49 (20.4)	0.11
≤ 40 years	75	9 (12.0)			22 (29.3)	
Angiographic subtype^b^						
Type I	130	17 (13.1)	0.005	0.43 (0.23 to 0.78)	28 (21.5)	0.78
Type Iia	21	8 (38.1)			6 (28.6)	
Type Iib	9	3 (33.3)			2 (22.2)	
Type III	13	5 (38.5)			3 (23.1)	
Type IV	15	3 (20.0)			1 (6.7)	
Type V	142	34 (23.9)			35 (24.6)	
Surgery (all)						
Present	66	9 (13.6)	0.092	0.51 (0.24 to 1.09)	13 (19.7)	0.62
Not present	246	58 (23.6)			57 (23.2)	
Surgery after IS treatment						
Present	36	7 (19.4)	1.0		9 (25)	0.67
Not present	273	59 (21.6)			60 (22.0)	
Refractory disease						
Refractory	114	31 (27.4)	0.084	1.68 (0.97 to 2.93)	20 (17.5)	0.12
Not refractory	193	35 (18.1)			49 (25.4)	

^a^HC, healthy control; IS, immunosuppressive; TAK, Takayasu's arteritis. Subgroup analysis was performed between groups with or without known clinical features. ^b^*P *vs other all other subtypes.

## Discussion

Researchers in various previous studies have demonstrated a strong association of HLA-B*52 in TAK. Our first study from Turkey, with a relatively large sample, has confirmed this association. No association of HLA-B*51 with TAK was present in this series.

The highest presence of HLA-B*52 in TAK was previously reported in a Japanese population (41%, RR = 2.2) [11]. In Japan, HLA-B*52 is also associated with aortic insufficiency, ischemic heart disease and pulmonary involvement [21]. We observed no association with these clinical features in our series. However, the routine use of ISs in our patients might have affected our results. Features of a more severe and refractory disease are also associated with HLA-B*52 presence in Japan: higher blood pressure, acute phase response and corticosteroid requirement [22,23]. Interestingly, we also observed a mild association of HLA-B*52 with more extensive aortic disease and refractoriness to treatment, which might suggest an association of HLA-B*52 with a more severe disease spectrum.

Studies from East Asian countries such as Korea, Thailand and India confirmed a HLA-B*52 association with TAK, though usually with a lower prevalence than Japan (Thailand and Korea both 15%) [12,24]. The lower presence of HLA-B*52 in patients with TAK might be associated with the lower prevalence in the background HCs (HLA-B*52 in HCs in Japan 23%, in Thailand 2.3% and in Turkey 6.7%). Association of HLA-B*52 with TAK has also been reported in other ethnic groups, such as Mexicans and Greeks [25,26]. Lack of association in North America and Arab populations requires confirmatory studies, as the number of samples has been too low in these studies to draw any conclusions [13,14].

Late-onset TAK (> 40 years of age) poses difficulties in the differential diagnosis from giant cell arteritis (GCA), and the two diseases are suggested to be a continuum with overlapping features [27]. In this context, HLA-B*52 might be a specific genetic susceptibility factor for classic early-onset TAK, as no association of GCA with HLA-B*52 has been reported previously [28].

Associations with other HLA genes in patients from Korea (HLA-A*3001, HLA-DRB1*1502) and Mexico (HLA-DRB1*1301) have also been reported; however, they have not been confirmed in other populations [29,30]. Although regions close to *HLA-B*, such as major histocompatibility complex class I-related chain A, have also been investigated for linkage disequilibrium in Japan, no further association has been confirmed [31]. An association of HLA-B*3901 with TAK has also been reported in studies conducted in Japan and Mexico [32,33]. However, this association has not been confirmed in India, Greece or Korea [17,26,29]. We also previously looked at HLA-B*39 in a subset of our population and observed no association with TAK (2.9% vs. 3.6%) (unpublished observation, M. Bicakcigil, et al).

The lack of association of HLA-B*51 with TAK, a granulomatous vasculitis, has important implications for the pathogenesis of TAK. As HLA-B*51 (a very common allele in Turkey) is associated only with Behçet's disease, an inflammatory disease involving the activation of both innate and adaptive immunity, peptide-binding differences of HLA-B*51 and HLA-B*52 seem to predispose patients to very different clinical phenotypes of vasculitis [34]. Moreover, the association of HLA-B*52 seem to be weaker in Turkish TAK patients compared to the HLA-B*51-Behçet's association, suggesting that other genetic factors might have a larger effect on disease susceptibility in TAK [35].

Our study has some limitations. All patients were followed in tertiary centers and may reflect a more severe disease spectrum. As TAK is a rare disease, however, we think that most patients suspected or diagnosed as having TAK are referred to specialized centers in Turkey. The gender ratio among TAK patients and HCs was not well-matched in our study. Finally, we chose to study only the *HLA-B *types previously associated with TAK, and other alleles need to be studied as well.

## Conclusion

We have confirmed the association of the HLA-B*52 allele with TAK in patients in Turkey. The negative association with late-onset and milder forms of the disease needs to be confirmed in other populations.

## Abbreviations

GCA: giant cell arteritis; HC: healthy control; HLA: human leukocyte antigen; IS: immunosuppressive; NK: natural killer; PCR: polymerase chain reaction; SNP, single-nucleotide polymorphism; SSP: sequence-specific primer; TAK: Takayasu's arteritis; TNF, tumor necrosis factor.

## Competing interests

The authors declare that they have no competing interests.

## Authors' contributions

ZS participated in patient data collection, performed genotyping, interpreted and analyzed the data and wrote the manuscript. MB participated in design of the study, sample and data collection and genotyping. KA, SK, SA, FO, OK, ZO, AA, HTEO, ES, MAO, AC, VC, AMO, ET, ND, SZA, NY, IF, YK, SK NA, MI and GK participated in sample and data collection from the patient group, interpretation of the data and manuscript preparation. VY and FAU participated in sample and data collection from healthy controls and in genotyping. HD and GSD designed and coordinated the study, analyzed and interpreted the data and wrote the manuscript. All authors read and approved the final manuscript for publication.
